# The Impact of Night Shifts, Tobacco Dependence, Health Awareness, and Depression Risk on Chronic Disease Risk Among Generation Z Overtime Workers During the COVID-19 Pandemic

**DOI:** 10.3390/healthcare13050569

**Published:** 2025-03-06

**Authors:** Hui-Li Lin, Wen-Hsin Liu

**Affiliations:** 1 Department of Tourism Management, Nanhua University, Chiayi 622301, Taiwan; linsun3742@gmail.com; 2 Division of Family Medicine, Ditmanson Medical Foundation Chia-Yi Christian Hospital, Chiayi 600566, Taiwan

**Keywords:** Generation Z, overtime workers, night shifts, tobacco dependence, depression, chronic disease risk, health awareness, mediated effect

## Abstract

**Background/Objectives**: the COVID-19 pandemic accelerated the adoption of remote work, blurring the boundaries between professional and personal life. This shift resulted in longer working hours, negative emotional outcomes, and health issues, particularly among Generation Z employees. This study investigates the links between working overtime, tobacco dependence, night shifts, and chronic disease risk in Generation Z employees during the pandemic while also examining the roles of depression risk and health awareness. A quantitative research approach was used to administer a questionnaire and employ the chi-square test, *t*-test, and logistic regression analysis to compare overtime-related factors and chronic disease risks. **Results**: the overtime workers are 1.39 times more likely to develop chronic diseases than those who do not work overtime. The odds ratio (OR) for overtime workers is 1.41, indicating that working overtime is a major risk factor for chronic disease. Among overtime workers, tobacco dependence and depression risk are significantly correlated with the risk of chronic disease, while night shift work is not. Overtime workers’ health awareness is significantly correlated with chronic disease risk and has a partial mediating effect on the relationship between tobacco dependence and chronic disease risk. This is due to the strong correlation (*p* < 0.001) between tobacco dependence and chronic disease, which limits the extent to which health consciousness can mitigate the negative effects of tobacco dependence. **Conclusions**: these findings highlight the importance of smoking cessation and mental health interventions in reducing the risk of chronic disease for Generation Z workers, particularly in the post-pandemic era.

## 1. Introduction

Although previous studies have conducted meta-analyses on long working hours, working overtime, and occupational health [1,2], there are limitations in the relevance of these meta-analyses to the study of the health of young workers in the context of the COVID-19 pandemic due to the adoption of widespread remote and home-based work during the pandemic. In the post-pandemic era, remote work has evolved from a preventive measure to a new workplace model adopted by many organizations. This shift has not only redefined the boundaries of time and space for work but also blurred the lines between employees’ work and personal lives, leading to negative emotional responses and physical and mental health issues [3,4]. Research indicates that this constant work-ready lifestyle significantly impacts the health of young Generation Z employees [5,6,7]. Generation Z refers to individuals born approximately between 1990 and 2010 [8], a cohort that grew up with internet technology and digitalization [8,9,10]. They are the most globally connected and highly educated generation [11]. By 2025, Generation Z is projected to comprise nearly 30% of the global workforce [12], making them the largest professional demographic worldwide. While members of Generation Z benefit from technological advancements and possess superior information-processing abilities, they face challenges due to a rapidly changing environment, including the occurrence of global pandemics and record-breaking inflationary pressures [12]. This generation demonstrates high work engagement but is also subject to the challenges of long working hours. Prolonged work hours have not only increased their stress and depression risk beyond those of previous generations [7,13] but are also strongly associated with various chronic diseases, such as cardiovascular conditions, diabetes, and mental health disorders [2,14,15]. Additionally, behaviors like smoking and working night shifts pose further health risks to Generation Z. In 2019, the prevalence of a previous cigarette smoking habit among young adults ranged from 13.6% in those with any chronic disease to 20.0% in those with two or more chronic diseases [16]. Epidemiological studies suggest that smoking significantly increases the risk of cardiovascular disease in young individuals [17,18]. Both the quantity and duration of smoking elevate this risk, particularly when smoking begins during adolescence, with its health impacts being especially pronounced in young adults [19]. For Generation Z, although the prevalence of tobacco dependence has declined [16,20], some young individuals still rely on tobacco to cope with work-related stress, using smoking as a strategy to manage increasing pressure [21] and thereby exacerbating their health risks. For instance, one study found that overtime workers experience higher smoking rates due to the heavy physical and mental load of work, with smokers facing more than twice the risk of cardiovascular disease compared to non-smokers [22]. Smoking has been identified as a potential mediating factor in the causal relationship between long working hours and cardiovascular disease [23,24,25,26,27]. In addition, night shift work disrupts the body’s circadian rhythm, posing significant health risks. Studies have identified shift work and night shifts as some of the most harmful work schedules for employees [28,29,30]. These schedules negatively impact physical and mental health, family and social life, and organizational environments. Long-term night shift workers are also at a higher risk of developing chronic conditions such as obesity, diabetes, and cardiovascular diseases [31].

The health impacts of prolonged overtime extend beyond physical effects to psychological ones. Work-related stress due to overtime is considered a major factor contributing to the risk of depression [32]. Stress alters hippocampal neuroplasticity, which is closely linked to the pathogenesis of depression [33], establishing a strong connection between work stress and increased depression risk [34,35,36,37]. Employees experiencing chronic work stress are more likely to exhibit depressive symptoms [31]. These symptoms can accelerate the development of chronic conditions such as cardiovascular diseases and diabetes, exacerbate chronic pain, and create a vicious cycle of mutual reinforcement [38,39,40], negatively affecting both personal work performance and overall quality of life.

To address the negative health impacts of overtime, shift work, and smoking among Generation Z workers, enhancing health awareness—which is defined as an individual’s ability to recognize and perceive their own health status—has been identified as a crucial factor for preventing disease and promoting healthier behaviors [41,42]. Health awareness functions as a psychological and behavioral regulatory mechanism [43,44], potentially encouraging overtime workers to adopt health-promoting behaviors, thereby reducing the incidence of chronic disease. Studies suggest that employees with greater health awareness are better equipped to manage stressors [45,46] and are more likely to engage in proactive health behaviors [45], which can lower the risk of depression and further mitigate chronic disease risks. Additionally, individuals with good health awareness are more likely to participate in regular health check-ups, facilitating the early detection of potential chronic disease [46]. Enhancing health awareness, particularly among individuals engaged in long-term overtime or night shifts, may encourage healthy behaviors, such as smoking cessation, thereby reducing the risks of depression and chronic disease. Understanding the unhealthy behaviors and lifestyles of Generation Z overtime workers could contribute to lowering chronic disease risk. Moreover, analyzing health awareness among overtime workers can provide positive insights for chronic disease prevention and health improvement. However, research on the interactions between these factors and the risk of chronic disease among Generation Z overtime workers remains limited. Therefore, the purpose of this study is to explore the relationship between tobacco dependence, night shift work, and chronic disease risk among Generation Z overtime workers. It further analyzes how psychological factors such as depression risk and health awareness influence chronic disease risk and examines the interactions between these factors in the risk of chronic disease for overtime workers. The aim is to reveal the significant impacts of overtime work, tobacco dependence, depression risk, and health awareness on the risk of chronic disease among Generation Z workers. The proposed findings enhance our understanding of the potential risks and protective factors associated with the impact of overtime work culture on employee health during the pandemic. They also provide a foundation for future health intervention strategies aimed at Generation Z workers who engage in overtime work, particularly in the post-pandemic era.

Based on the aim of this study, the following hypotheses are formulated:

**Hypothesis 1:**  *The risk of developing a chronic disease is higher among Generation Z employees who work overtime than among those who do not.*

**Hypothesis 2:**  *Among Generation Z workers, night shifts and tobacco dependence are significantly related to chronic disease.*

**Hypothesis 3:**  *There are significant differences in Generation Z overtime workers’ health awareness and risk of depression in relation to chronic disease.*

**Hypothesis 4:**  *Tobacco dependence, night shifts, health awareness, and depression risk all affect the risk of chronic disease in Generation Z overtime workers.*

**Hypothesis 5:**  *The health awareness of Generation Z overtime workers has a significant mediation effect on tobacco dependence and chronic disease.*

## 2. Methods

### 2.1. Participants and Procedure

The data used in this study are secondary data from the “Children and Adolescent Behaviors in Long-term Evolution” project (C00348) in the Survey Research Data Archive of the Academia Sinica [47]. This project has been approved by the Institutional Review Board (IRB) of the National Health Research Institutes (NHRI) in Taiwan, with approval number EC9009003. Project C00348 focused on Taiwan’s Generation Z workers (individuals born between 1990 and 1995). The data processing adhered to the ethical protocol of Academia Sinica and complied with data protection policies. Furthermore, no personal identifiers were present in the dataset, ensuring that the research adhered to ethical standards and maintained confidentiality. The survey instruments demonstrated high reliability and validity. Random sampling was used to conduct the survey during the COVID-19 pandemic. Respondents were first contacted via email to obtain their consent before telephone interviews were conducted. In total, there were 2771 participants. After excluding incomplete or invalid responses, 1984 valid questionnaires were retained.

Key demographic statistics showed that 36.84% of the respondents were overtime workers, and 63.16% were non-overtime workers. Among the participants, 5.61% reported tobacco dependence and 94.39% did not; 13.00% worked night shifts, and 87.00% did not. The level of education was distributed as follows: 20.61% held a master’s degree or higher, 73.08% had a university degree, and 6.3% had a high school diploma or lower. Regarding marital status, 91.63% were unmarried, and 8.37% were married. Occupation types included management (2.42%), professionals and technical workers (30.29%), clerical staff (34.43%), service industry workers (13.76%), manual laborers (3.93%), military personnel (35%), and others (13.41%).

### 2.2. Measures and Reliability/Validity

This study invited three experts to review and select questionnaire items from the C00348 dataset that aligned with the research objectives. The selection process involved discussions to ensure the items were relevant and consistent with findings reported in the literature. The items selected focused on variables such as overtime work, night shifts, tobacco dependence, and chronic disease, which have been documented as interrelated [16,28,29,30]. The literature suggests that overtime work negatively impacts physical and mental health, family and social life, and organizational environments, and long-term night shift workers are at a higher risk of developing chronic diseases such as obesity, diabetes, and cardiovascular conditions [31]. The risk of depression has been shown to correlate with health outcomes [38,39,40], and health awareness is recognized as an important factor in promoting health [41,42].

After formal discussions and with the unanimous agreement of the expert panel, the questionnaire items to be analyzed were finalized. The selected items underwent expert review and were confirmed to have content validity. The questionnaire included items covering four dimensions, totaling 18 questions: demographic variables (education level, marital status, and occupational category), health awareness, health behaviors (overtime work, night shift work, and tobacco dependence), and health status (depression risk and chronic diseases).

Chronic diseases included hypertension, diabetes, and hyperlipidemia. Overtime work was defined based on Article 24, Paragraph 1 of the Labor Standards Act [48], which classifies working more than 40 h per week as overtime, while working equal to or less than 40 h is not considered overtime. Tobacco dependence was measured using the Nicotine Dependence Scale [49,50], with scores of 4 or above (equivalent to smoking more than 10 cigarettes daily) indicating moderate to severe nicotine dependence. A monthly consumption of 301 cigarettes or more was classified as tobacco dependence, while 300 cigarettes or fewer indicated no dependence.

Health awareness was measured on a 4-point Likert scale ranging from 1 (very unhealthy) to 5 (very healthy). Depression risk was assessed using the Taiwanese Depression Scale (TDS) [51], an official tool developed for individuals aged 18 and above in Taiwan. The TDS has been validated through large-scale implementation since 2004 and provides normative scores with established reliability and validity. The scale uses a 4-point Likert scale ranging from 1 (none or rarely) to 4 (always).

Reliability analysis for the 10 depression risk items evaluated in this study yielded a Cronbach’s alpha of 0.842, indicating high internal consistency. Deleting any item did not improve the original internal consistency coefficient (Cronbach’s alpha range: 0.812–0.842). Therefore, no items were removed, confirming that the depression risk items in this study exhibit high reliability.

### 2.3. Data Analysis

After confirming the validity and reliability of the research content, data analysis was conducted in two stages using SPSS 21.0. (1) A chi-squared test was used to examine the relationship between demographic variables, working overtime, and chronic disease, ensuring the results were scientifically objective and did not simply reflect specific population variables; if no significant link was found between chronic disease and demographic variables, Excel was used to calculate the chronic disease risk for those who did and did not work overtime. (2) Based on the finding that overtime workers have a higher chronic disease risk (with an odds ratio greater than 1), chi-squared tests and independent sample *t*-tests were used to identify relationships and differences among key variables in the overtime group. Finally, binary logistic regression was used to explore how key overtime group-related variables affect chronic disease risk and their potential mediating effects. The methods described below were applied.

(1) Chi-squared test:
  (a) Used to analyze the associations between demographic variables (marital status, education level, and occupational category) and overtime work.
  (b) Used to investigate the relationships between demographic variables and chronic diseases.
  (c) Used to examine correlations among key categorical variables within the group working overtime.
(2) Independent sample *t*-test:
  Used to compare differences in depression risk and health awareness between participants with and without chronic disease in the overtime group.
(3) Chronic disease risk calculation:
  Excel was used to calculate the risk of chronic disease among participants working overtime.
(4) Binary logistic regression:
  (a) Used to control for demographic variables to analyze the effects of night shifts, tobacco dependence, depression risk, and health awareness on chronic disease within the overtime group.
  (b) Used to examine the mediated effects of these variables on the risk of chronic disease.

## 3. Results

This study found that overtime workers have a higher risk of chronic disease than non-overtime workers, with an odds ratio (OR) of 1.41, confirming working overtime as a risk factor for the development of chronic disease. To further understand the influencing factors, we analyzed the impact and mediating effects of key variables on chronic diseases in the overtime group. The results are as follows.

### 3.1. Analysis of Demographic Variables, Working Overtime, and Chronic Diseases Among Generation Z Employees

The data from 1984 valid questionnaires were analyzed using the chi-squared test. The results indicated that employees who do not work overtime (1253 people) account for 63%, while overtime workers (731 people) account for 37%, indicating that more than one-third of participants work overtime. Among the workers, 95% (1897 people) do not have a chronic disease, while 5% (87 people) do, suggesting that a significant proportion of workers are affected by chronic conditions. Among the demographic variables, the relationships between overtime work and education level and between overtime work and marital status were examined and found to be insignificant (*p* > 0.05), while the relationship between occupation type and overtime work was significant (*p* ***). Although the analysis results show that overtime work is significantly related to occupation type, chronic diseases are not significantly related to demographic variables (*p* > 0.05) (as shown in Table 1). Therefore, further analysis was conducted to assess the risk of chronic disease in workers, controlling for demographic variables. The sample was divided into two groups based on working hours: a treated group (TG), which included those working overtime, and a control group (CG), which included those not working overtime, as shown in Table 2. Subsequent analyses focused on evaluating the risk of chronic disease among workers in these two groups.

Table 2 reveals that the prevalence rate (PR) of chronic diseases is higher in the treated group (TG) compared to the control group (CG), with values of 0.0534 and 0.0383, respectively. The relative risk (RR) of chronic diseases for the TG compared to the CG is 1.39. This indicates that workers in the TG are 1.39 times more likely to develop chronic diseases than those in the CG.

The odds ratio (OR) for the treated group (TG) is 1.41, which is greater than 1, with a 95% confidence interval of (0.92, 2.18). An odds ratio (OR) greater than 1 indicates a risk factor, while an OR of less than 1 represents a protective factor. This result suggests that overtime work is a harmful factor in the occurrence of chronic diseases. Therefore, further analysis of the associations and regression models for key variables in the TG is warranted to better understand these relationships.

### 3.2. Correlation Analysis of Key Variables Among Generation Z Overtime Workers

This study analyzed 731 overtime workers, among whom 731 work overtime, 39 (5.3%) have chronic disease, 41 (6%) have tobacco dependence, and 95 (13%) work night shifts. Additionally, 10 (1.3%) have both chronic disease and tobacco dependence, 4 (0.5%) have both chronic disease and work night shifts, and 8 (1.1%) have tobacco dependence and work night shifts. The chi-squared test results for the key variables are as follows: (1) Smoking and chronic disease: *p* *** < 0.01, indicating a significant correlation. (2) Night shifts and smoking: *p* > 0.05, showing no significant correlation. (3) Night shifts and chronic disease: *p* > 0.05, showing no significant correlation. These results suggest that smoking is significantly associated with chronic disease, while night shifts do not show a significant relationship with either smoking or chronic disease (see Table 3).

### 3.3. Difference Analysis of Health Awareness and Depression Risk Among Overtime Workers with and Without Chronic Disease

The results of the independent sample *t*-test showed significant differences in health awareness (*p* ** = 0.006 < 0.01) and depression risk (*p* * = 0.036 < 0.01) between those who worked overtime and had chronic disease and those who did not. The health awareness of those with chronic disease (mean = 3.128) is lower than that of those without chronic disease (mean = 3.645), while the depression risk of those with chronic disease (mean = 2.05) is higher than that of those without chronic disease (mean = 1.87) (as shown in Table 4).

### 3.4. Regression Analysis of Main Variables of TG Group on Chronic Diseases

Through chi-squared tests and independent sample *t*-tests, it was found that tobacco dependence is significantly associated with chronic disease (*p* ***), and there are significant differences in health awareness (*p* **) and depression risk (*p* *) between those with and without chronic disease. Night shifts and chronic disease (*p* > 0.05) showed no significant association. However, potential confounding effects were considered. This study used two categorical independent variables (tobacco dependence and shift work) and two scalar independent variables (health awareness and depression risk) to predict the risk of chronic disease among overtime workers and analyze the possibility of confounding variables. The results of the binary logistic regression analysis (Table 5) are provided for the following models: (1) Night shift work predicts chronic disease in overtime workers—*χ*^2^ = 0.291, df = 2, *p* > 0.05, Wald test *p* > 0.05 (Table 5). This shows that working night shifts has no predictive power for chronic disease in overtime workers, and the impact is not significant. (2) Tobacco dependence predicts chronic disease in overtime workers—*χ*^2^ = 18.345, df = 1, *p* ***: R^2^ = 0.073. This means that the model is predictive and has an explanatory power of 0.073, and the accuracy of the prediction value is 94.7%. That the Wald test is *p* *** (Table 5) shows that tobacco dependence has a significant impact on chronic disease in overtime workers. (3) Health awareness predicts chronic disease in overtime workers—*χ*^2^ = 11.732, df = 1, *p* **; R^2^ = 0.047. This means the model is predictive, with a prediction accuracy of 94.7%, and has an explanatory power of 0.047. That the Wald test is *p* ** (Table 5) shows that health awareness has a significant impact on chronic disease in overtime workers. (4) Depression risk predicts overtime workers’ chronic disease—*χ*^2^ = 4.091, df = 1, *p* *; R^2^ = 0.006. This means the model is predictive, with a prediction accuracy of 94.7%, and has 0.006 explanatory power. That the Wald test is *p* * (Table 5) shows that the risk of depression has a significant impact on chronic disease in overtime workers. (5) Using shift work, tobacco dependence, health awareness, and depression risk as covariates to predict chronic diseases resulted in *χ*^2^ = 28.180, df = 4, *p* ***; R2 = 0.038. This indicates that the model has predictive power with an explanatory value of 0.038, and the prediction accuracy is 94.7%. The Wald test shows that shift work is *p* = 0.505 > 0.05, depression risk is *p* = (0.267) > 0.05, tobacco dependence is *p* *** (0.000) < 0.001, and health awareness is *p* * (0.014) < 0.05 (Table 5). This suggests that shift work and depression risk have no significant effects on chronic disease, while tobacco dependence and health awareness have significant effects.

### 3.5. Mediating Effect of Health Awareness Between Tobacco Dependence and Chronic Disease

In this study, the mediating effect of chronic disease was tested using three models: (1) a predictive model of the effect of health awareness on chronic disease, (2) a predictive model of the effect of tobacco dependence on chronic disease, and (3) a predictive model of the effect of tobacco dependence and health awareness as covariates on chronic disease. These models satisfy the following four conditions: (1) Tobacco dependence (IV) has a significant effect on chronic disease (DV). (2) Health awareness (M) has a significant effect on chronic disease (DV). (3) Tobacco dependence (IV) has a significant effect on health awareness (M). (4) Tobacco dependence (IV) and health awareness (M) simultaneously have significant effects on chronic disease (DV). The first and second conditions were confirmed as stated above (Table 5). The third condition was verified through regression analysis between tobacco dependence and health awareness, yielding *χ*^2^ = 9.278, df = 1, and *p* **: R^2^ = 0.036. This indicates that the model has predictive power with an explanatory value of 0.036 and an accuracy of 94.4%. The regression analysis results (B = −0.525, S.E. = 0.170, Wald = 9.5, df = 1, *p* ** = 0.002) and 95% confidence interval (0.424, 0.826) demonstrate a significant relationship between tobacco dependence and health awareness. It can be inferred that health awareness may mediate the relationship between tobacco dependence and chronic disease.

**Figure 1:**
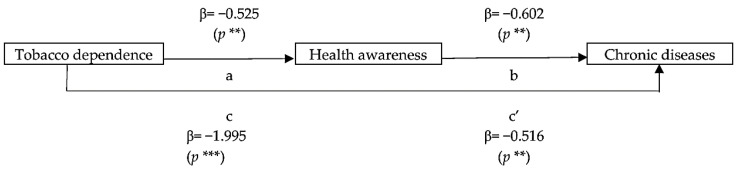
Health awareness (M) demonstrates a partial mediating effect on the influence of tobacco dependence on chronic disease. *p* **: *p* < 0.01; *p* ***: *p* < 0.001.

After confirming the first three conditions, the mediating effect was verified to satisfy the fourth condition. In this study, taking health awareness (M) as the mediating variable, the model results were *χ*^2^ = 26.514, df = 2, *p* ***, R^2^ = 0.105, indicating that the model has predictive power, with an explanatory value of 0.105 and an accuracy of 94.7%. The regression coefficients showed health awareness B = −0.516 (*p* **) and tobacco dependence B = −1.800 (*p* ***) (Table 5). These results satisfy the fourth condition, which states that tobacco dependence (IV) and health awareness (M) both have a significant impact on chronic disease (DV). This demonstrates that health awareness (M) partially mediates the effect of tobacco dependence on chronic disease (*p* **) (as shown in Figure 1), confirming that health awareness has a partial mediating effect.

## 4. Discussion

**Figure 2:**
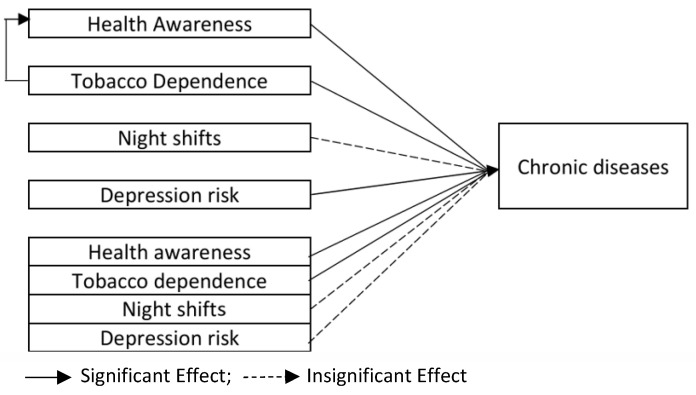
Relationship of main variables affecting risk of chronic disease among Generation Z overtime workers.

The results of this study indicate that working night shifts, whether analyzed as a single variable or in combination with other key variables (tobacco dependence and depression risk), does not affect the risk of chronic disease among Generation Z overtime workers. This confirms that working night shifts is not a significant factor influencing chronic disease in the study population. Depression risk and tobacco dependence have harmful effects on the occurrence of chronic disease among Generation Z workers, while health awareness has a positive impact and demonstrates a partial mediating effect between tobacco dependence and chronic disease. The interaction effects of key variables on chronic diseases are illustrated in Figure 2. These findings were validated through discussions with three domain experts and are supported by the relevant literature, as shown below.

(1) The Harmful Effects of Working Overtime on the Risk of Chronic Disease Among Generation Z Workers

Overtime workers have a higher risk of developing chronic disease compared to those who do not work overtime (OR = 1.41), indicating that overtime tends to have harmful effects on health. This finding aligns with the existing literature, which reports that long working hours increase the risk of various chronic diseases, particularly cardiovascular diseases, metabolic syndrome, and diabetes [23,52,53,54]. Moreover, prolonged working hours can negatively affect overall health. Implementing flexible work schedules may help alleviate fatigue and improve work–life balance, potentially mitigating the risk of chronic disease among Generation Z workers [55].

(2) The Insignificant Effect of Working Night Shifts on Chronic Disease Among Generation Z Overtime Workers

Regression analyses examining working night shifts, smoking habits, health perception, and depression risk revealed that working night shifts, whether as a standalone independent variable or combined with others (smoking habits, health perception, and depression risk), had no significant effect on the risk of chronic disease. This suggests that taking night shifts does not influence overtime workers’ risk of developing chronic diseases. Through expert discussion, this study inferred that Generation Z workers’ risk of chronic disease would not be affected by whether they work night shifts or not. This may involve physiological factors. According to some studies, young people have higher hormone regulation ability, such as melatonin secretion, which helps alleviate the physiological burden of night shift work [56]. Younger people are better able to regulate their physiological hormones, which may also help reduce the risk of chronic disease caused by night shift work and reduce the negative health impact of night shift work. Additionally, many modern workplaces, particularly in high-tech industries, have gradually improved shift schedules by implementing more scientifically designed shift systems, thereby reducing the long-term physiological effects of night work [57]. These improvements are particularly effective for younger workers, further lowering the risk of chronic disease.

(3) The Significant Effect of Depression Risk as a Single Independent Variable on Chronic Disease Among Generation Z Overtime Workers

The findings indicate that depression risk, as a single independent variable, significantly impacts chronic disease risk. Overtime workers with chronic diseases exhibit a higher depression risk (mean = 2.05) than those without chronic diseases (mean = 1.87). This aligns with prior studies reporting that overtime work harms both physical and mental health. Individuals working more than 40 h per week, particularly in high-stress environments, exhibit a higher prevalence of depression, with a stronger association between extended working hours and mental health issues [58].

(4) The Partial Mediating Effect of Health Perception Between Smoking and Chronic Diseases Among Generation Z Overtime Workers

Both smoking and health perception, as independent variables, significantly influence chronic disease (*p* < 0.05). This supports the inference that “the presence of chronic diseases” is associated with “smoking” and “health awareness”. Specifically, smoking shows an Exp(B) = 0.165, or OR = 0.165, indicating that individuals who do not smoke (coded as 0) are 0.165 times as likely to develop chronic diseases compared to smokers (coded as 1). The conclusion that smoking has a harmful effect is consistent with related research findings. Smoking is a significant risk factor for chronic disease and poses considerable harm to health [59]. It substantially increases the risk of cardiovascular disease among younger individuals, with smokers exhibiting much higher rates of cardiovascular disease compared to non-smokers [17,18].

Health awareness shows Exp(B) = 0.597, or OR = 0.597 < 1, indicating that for every one-point increase in health perception, the likelihood of having a chronic disease (0.597) is 0.403 times lower than the likelihood of not having a chronic disease (1). This suggests that health awareness has a protective effect. Smoking directly influences chronic disease, with Exp(B) = 0.136. When health awareness serves as a mediating factor, the influence of smoking on chronic disease changes to Exp(B) = 0.165. This indicates that under the mediating effect of health perception, the likelihood of smokers developing chronic diseases compared to non-smokers decreases from 7.35 times (1/0.136) to 6.06 times (1/0.165). Thus, health awareness is a mediating factor that reduces the OR of smokers developing chronic diseases. Health awareness acts as a protective factor for chronic disease risk among smokers who work overtime. Even with the mediating effect of health perception, the influence of smoking on chronic disease remains significant (Wald: *p* *** → *p* ***). This confirms that health awareness has a partial mediating effect. Smoking status among overtime workers affects chronic disease risk, and health awareness also exerts a notable influence on the relationship between smoking and chronic disease.

Overtime work affects the risk of chronic disease among Generation Z workers, and smoking undoubtedly increases this risk. However, health awareness plays a mediating role in reducing chronic disease risk. Health awareness encourages individuals to pay more attention to their health, thereby reducing the harm caused by unhealthy habits, particularly among smokers [60]. Studies have shown that health awareness significantly predicts smoking behavior [60,61]. When an individual’s awareness of health improves, they are more likely to adopt healthier behaviors and smoke less. This change may occur due to the psychological mechanism of “self-awareness”, where smokers who understand the harmful effects of smoking are more motivated to quit [62]. While health awareness positively mediates health behaviors and reduces chronic disease risk, the negative health impact of smoking remains significant. This finding highlights the profound physiological impact of smoking, which cannot be fully mitigated by merely improving health awareness.

According to the relevant literature, tobacco dependence may arise from a dual dependency on psychological and physiological factors [63]. Smoking often serves as a form of emotional regulation, with smokers potentially struggling to effectively cope with emotional or stress-related issues effectively. At the same time, long-term smokers may develop a physiological dependency on tobacco, diminishing their sensitivity to health concerns and reducing their awareness of the risk of chronic disease. Even when smokers recognize the harmful health effects of smoking, physiological or emotional dependencies may prevent them from altering their behavior, leaving them vulnerable to high-risk health conditions. Thus, while health perception plays a positive mediating role in promoting healthy behaviors and improving health outcomes, the negative health impact of smoking remains significant. Given the strong connection between smoking and chronic disease risk, smoking cessation interventions and psychological support are critical in mitigating the adverse effects of smoking. Relying solely on enhancing health perception is insufficient to eliminate the health risks associated with tobacco dependence.

Pre-pandemic studies may have limitations in addressing the work-related stress and health impacts of the pandemic. Therefore, this study provides valuable insights for medical staff in the post-pandemic era to develop smoking cessation programs for Generation Z overtime workers, focusing on stress-related smoking patterns, and to design mental health screening and intervention protocols based on the link identified between depression risk and chronic disease.

## 5. Conclusions

In summary, Generation Z workers who work overtime have an odds ratio (OR) greater than 1 for developing chronic diseases, confirming that overtime is a risk factor for such conditions. Adjusting overtime policies and improving work environments may contribute to better overall health for Generation Z employees. For this group, shift work (working night shifts) shows no significant relationship with chronic disease risk and is not a risk factor for developing chronic disease. Smoking and the risk of depression independently and significantly affect chronic disease risk among Generation Z workers, and both have a harmful impact. In particular, the strong association between smoking and chronic disease underscores the importance of smoking cessation. Health awareness plays a partial mediating role in the chronic disease risk of smokers. For overtime workers who smoke, greater health awareness can increase the perception of their health status and encourage healthier behaviors, serving as a protective factor. However, the physiological and psychological dependency inherent in smoking limits the mitigating effects of health awareness. Regardless of health awareness, smokers remain at a higher health risk due to their dependence on smoking. Therefore, although improving health awareness helps reduce the negative impact of smoking on chronic disease risk, smoking cessation interventions and psychological support remain essential measures for reducing chronic disease risk among Generation Z overtime workers.

## 6. Limitations and Future Research

The sample was limited to Generation Z Taiwanese workers born between 1990 and 1995, which may limit the generalizability of the results. Future research should aim to replicate this study in different geographical regions and age subgroups within Generation Z. It may also be valuable to explore other potential factors that may interact with the variables examined in this study, such as diet and exercise habits.

## Figures and Tables

**Table 1:** Demographic characteristics of Generation Z, the association between overtime work and chronic diseases.

Demographic Variables	Does the Participant Work Overtime?			Does the Participant Have Chronic Disease?		
	No	Yes	Sig. (2-Tailed)	No	Yes	Sig. (2-Tailed)
Education level: master’s degree or above	273	136	Value = 3.71 df = 2 *p* > 0.05	388	21	Value = 3.62 df = 2 *p* > 0.05
College	907	543		1393	57	
High school and below	73	52		116	9	
Marital status Has no spouse	1147	671	Value = 0.04 df = 2 *p* > 0.05	1739	79	Value = 0.08 df = 2 *p* > 0.05
Has a spouse	106	60		158	8	
Occupation Manager	18	30	Value = 215.99 df = 6 *p* ***	46	2	Value = 6.99 df = 6 *p* > 0.05
Professional/technical occupation	323	278		570	31	
General Affairs	447	236		662	21	
Services	140	133		258	15	
Laborer	49	29		73	5	
Others	264	2		253	13	
Military	12	23		35	0	
Total number of participants	1253	731		1897	87	

*p* ***: *p* < 0.001.

**Table 2:** Generation Z overtime workers with chronic illness.

Group	Chronic Illness	No Chronic Illness	N Total
Working overtime (TG)	39 people	692 people	731 people
Not working overtime (CG)	48 people	1205 people	1253 people
Total number of participants	87 people	1897 people	1984 people

**Table 3:** Chi-squared test results for key variables in overtime workers.

		Smoking			Night Shifts					Night Shifts		
		No	Yes	N Total	No	Yes	N Total			No	Yes	N Total
Participant has a chronic disease?	No	661	31	692	601	91	692	Smoking	No	603	87	690
	Yes	29	10	39	35	4	39		Yes	33	8	41
n total		690	41	731	636	95	731	N total		636	95	731
Pearson *χ*^2^ = 31.228, df = 1, *p* *** (0.000) < 0.001					Pearson *χ*^2^ = 0.273, df = 1, *p* (0.601) > 0.05			Pearson *χ*^2^ = 1.631, df = 1, *p* (0.202) > 0.05				

Note: n = 731, *p* *** < 0.001 (2-tailed).

**Table 4:** Difference analysis of health awareness and depression risk among participants with chronic disease.

Group Statistics						Independent Sample Test								
Participant Has a Chronic Disease?		n	Mean	SD	SEM	Levene’s Test		*t*-Test for Equality of Means						
						F	Sig.	t	df	Sig. (2-Tailed)	MAD	SE	95% CI	
													Lower	Upper
Health awareness	No	692	3.65	0.86	0.03	17.43	0.000	2.870	40.66	0.006	0.516	0.180	0.15	0.88
	Yes	39	3.13	1.11	0.18									
Depression risk	No	692	1.87	0.49	0.02	3.35	0.068	2.099	729	0.036	−0.172	0.082	−0.33	3.35
	Yes	39	2.05	0.61	0.10									

Note: n = 731.

**Table 5:** Regression analysis between main variables and chronic diseases.

IV	β	S.E.	Wald	df	Sig.	Exp(β)	95% EXP(β) CI	
							Lower	Upper
Night shifts	0.281	0.540	0.272	1	0.602	1.325	0.460	3.815
Constant	−3.125	0.511	37.407	1	0.000	0.044		
Smoking	−1.995	0.410	23.656	1	0.000	0.136	0.061	0.304
Constant	−1.131	0.364	9.679	1	0.002	0.323		
Health awareness	−0.602	0.174	11.928	1	0.001	0.548	0.389	0.771
Constant	−0.832	0.576	2.085	1	0.149	0.435		
Depression risk	0.624	0.300	4.340	1	0.037	1.866	1.038	3.357
Constant	−4.096	0.631	42.187	1	0.000	0.017		
Night shifts	0.372	0.558	0.444	1	0.505	1.450	0.486	4.330
Depression risk	0.359	0.324	1.231	1	0.267	1.432	0.759	2.699
Smoking	−1.821	0.426	18.280	1	0.000	0.162	0.070	0.373
Health awareness	−0.456	0.186	5.980	1	0.014	0.634	0.440	0.913
Constant	−0.436	1.040	0.175	1	0.675	0.647		
Smoking	−1.800	0.421	18.239	1	0.000	0.165	0.072	0.378
Health awareness	−0.516	0.179	8.297	1	0.004	0.597	0.420	0.848
Constant	0.454	0.647	0.494	1	0.482	1.575		

IV: Main variables affecting chronic diseases.

## Data Availability

The original data described in this study are available from the Survey Research Data Archive, Academia Sinica, accessible by application.
